# Endocarditis following Consumption of Cereal Associated with *Salmonella enterica* Subtype Mbandaka Outbreak

**DOI:** 10.1155/2019/5464230

**Published:** 2019-03-27

**Authors:** Jana K. Dickter, LiYing Cai, David S. Snyder

**Affiliations:** ^1^City of Hope, Division of Infectious Diseases, Duarte, CA, USA; ^2^City of Hope, Division of Cardiology, Duarte, CA, USA; ^3^City of Hope, Department of Hematology and Hematopoietic Cell Transplantation, Duarte, CA, USA

## Abstract

A 69-year-old immunocompromised man developed mitral valve endocarditis due to *Salmonella enterica* serotype Mbandaka, contracted from the cereal outbreak. The patient had a history of HLA-matched related hematopoietic stem cell transplant with persistent graft-versus-host disease (GVHD). This case report discusses prior international outbreaks that occurred due to *Salmonella enterica* subtype Mbandaka, the risks of developing endovascular infections from salmonellosis, and persistent infections that may develop more frequently with *S. enterica* serotype Mbandaka. The patient received a six-week course of intravenous antibiotics and remains on oral suppressive antibiotics, with his length of therapy to be determined based on his GVHD treatment.

## 1. Background

Nontyphoidal *Salmonella* is the second most common cause of foodborne illness in the United States [1]. Immunocompetent persons typically have a self-limited enteritis, but immunocompromised patients in particular may be at risk for invasive infections. Endocarditis is a rare complication of infection.

*Salmonella enterica* serotype Mbandaka is a rare cause of foodborne illness, and one of the largest outbreaks was the multistate outbreak linked to cereal. To date, there have been 34 cases causing hospitalization [2], but there are no known reports of *S. enterica* serotype Mbandaka causing endovascular complications. We report a case of *S. enterica* serotype Mbandaka endocarditis associated with the cereal outbreak.

## 2. Case Report

A 69-year-old man presented on May 1, 2018, to a hospital with fevers and diarrhea. The patient had a complicated history, with cardiovascular problems including an abdominal aortic aneurysm, atrial septal defect status after Amplatzer occluder device placed in 2008, and sick sinus syndrome status post dual-chamber pacemaker placement in 2015.

The patient was diagnosed with primary myelofibrosis with JAK2 mutation in April 2015, treated with lenalidomide and prednisone which were tapered off, and his treatment was switched to ruxolitinib. In November 2016, his disease transformed to secondary acute myelogenous leukemia. He was treated initially with induction chemotherapy with 7 + 3 cytarabine and daunorubicin, and follow-up bone marrow biopsies showed no evidence of leukemia. He was subsequently treated with one course of consolidation chemotherapy with high-dose cytarabine. He ultimately underwent HLA-matched related hematopoietic stem cell transplant from his brother in February 2017. His conditioning regimen was reduced-intensity fludarabine and melphalan. His transplant course was complicated by culture-negative neutropenic fevers and neurotoxicity due to tacrolimus, so he was switched to mycophenolate for graft-versus-host disease (GVHD) prophylaxis. Follow-up bone marrow biopsy showed persistence of fibrosis, but was negative for leukemia, and DNA testing proved that he had over 99% donor DNA in the cell population.

His posttransplant course was complicated by mild GVHD involving the skin and colon diagnosed by sigmoidoscopy. He was treated with high-dose steroids, budesonide, and sirolimus, which were ultimately tapered off by the end of July 2017. However, he developed chronic GVHD involving his mouth in September 2017, which was treated with prednisone, sirolimus, and dexamethasone swish and spit. His prednisone dose was tapered to 30 mg daily by November 2017, and by January 2018, he was only taking prednisone 10 mg daily. By March 2018, it was decreased to 10 mg every other day.

In March 2018, he developed an acute right lower extremity deep vein thrombosis involving his common femoral, profunda, femoral popliteal, posterior tibial, and peroneal veins. He was treated with tissue plasminogen activator, heparin, and thereafter apixaban.

He was admitted in early May 2018 for a week at an outside facility with abdominal pain, diarrhea, and septic shock requiring admission to the intensive care unit, necessitating the use of pressors and stress-dose steroids. He also developed acute renal failure but never required hemodialysis. Both blood and stool cultures were positive for *S. enterica* serotype Mbandaka. This was believed to be associated with the multistate outbreak of infections linked to cereal. The patient reported that he ate an entire box of cereal within one week prior to becoming ill. Blood culture susceptibilities demonstrated that the organism was susceptible to ampicillin, ceftriaxone, ciprofloxacin, tetracycline, tigecycline, and trimethoprim/sulfamethoxazole.

He was treated with IV ceftriaxone and discharged on amoxicillin/clavulanic acid, completing a total of three weeks of antibiotics. However, about a week after stopping antibiotics, at the end of May 2018, he was readmitted with fevers and diarrhea. Blood cultures were negative, but stool cultures were again positive for *S. enterica* serotype Mbandaka. He was treated initially with intravenous antibiotics and was discharged on 28 days of trimethoprim/sulfamethoxazole 1 DS tab twice per day. While on antibiotics, his abdominal pain, fevers, and diarrhea resolved.

Approximately seven days after stopping his antibiotics, in early July 2018, he developed a third recurrence of fevers and diarrhea. Blood cultures returned negative, but stool cultures were again positive. He was admitted to the hospital for the third time, treated with intravenous antibiotics for 3 days and discharged home with a second 28-day course of trimethoprim/sulfamethoxazole. Stool studies were also positive for *Clostridium difficile*, so he received oral vancomycin for treatment. His diarrhea and abdominal pain again resolved. Soon after his discharge, his prednisone dose was decreased to 5 mg every other day, and he was maintained on sirolimus 1 mg daily.

**Figure 1:**
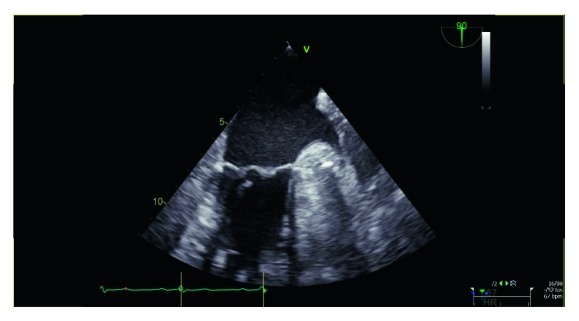
Transesophageal echocardiogram showing 0.9–1 centimeter mitral valvular vegetation.

After his third admission, he was evaluated by infectious diseases for his recurrent infections with salmonellosis. A transesophageal echocardiogram was performed, which showed a 0.9–1 centimeter mitral valvular vegetation (Figure 1). This appeared very mobile and was associated with mild eccentric mitral regurgitation. The Amplatz closure device showed no evidence of infection, and his pacemaker leads showed no vegetation.

The patient also had a CT scan of his abdomen which showed a mild increase in the degree of mural thrombus associated with the 2.9 × 2.7 cm calcified abdominal aorta in the lower abdomen, now measuring 10 mm compared to 7 mm on the prior exam from 14 months prior. An indium-111 white blood cell scan was negative.

The patient's antibiotics were converted from oral trimethoprim/sulfamethoxazole to ceftriaxone 2 grams intravenously daily for six weeks. He was maintained on oral vancomycin for his *Clostridium difficile* infection. Four weeks into intravenous therapy, however, he was rehospitalized again with fevers and diarrhea. Blood and stool cultures were negative, and stool for *Clostridium difficile* was negative. His fevers and diarrhea ultimately resolved, and he was discharged home.

A follow-up transesophageal echocardiogram prior to discontinuation of intravenous antibiotics showed a smaller linear echodensity, now 0.5 cm, attached to the anterior leaflet of the mitral valve. There continued to be mild mitral regurgitation. Again, both the Amplatz closure device and pacemaker leads showed no vegetation.

Upon completion of six weeks of intravenous antibiotics for endocarditis, the patient restarted trimethoprim/sulfamethoxazole. He was ultimately tapered off oral vancomycin for his infection with *Clostridium difficile*. Length of therapy for his salmonellosis is to be determined based on follow-up stool testing and immunosuppressive course.

## 3. Discussion

The Centers for Disease Control and Prevention estimates that there are approximately 1.2 million illnesses and 450 deaths annually in the United States due to nontyphoidal *Salmonella* [1]. The Foodborne Active Diseases Surveillance Network reported that the incidence of *Salmonella* infection in 2016 was 15.4 cases per 100,000 [3].

Since 2009, the U.S. National Library of Medicine has recorded 1079 isolates of *S. enterica* serotype Mbandaka sampled from both clinical and environmental specimens, from all over the world.

Isolates from the environment mainly come from animals known to be colonized with *Salmonella* species (poultry, eggs, cattle, and pork) but also from less commonly described animal sources, including catfish, crab, tilapia, octopus, reptiles, horses, sheep, goats, cats, dogs, and wolves. Other food sources have included fruits and vegetables, sesame seeds, peanuts, pistachios, flour, and various spices. Water and sewage sources have also been described [4].

On May 18, 2018, the U.S. Food and Drug Administration was notified about a cluster of *S. enterica* serotype Mbandaka illnesses in multiple states, and further investigation connected this infection to the cereal as a possible source of infection. As a result, the cereal was recalled the following month [5]. As of September 2018, there have been 135 people infected with the infection in 36 states, and 34 have been hospitalized [2].

*S. enterica* serotype Mbandaka has rarely been implicated in prior outbreaks. It was first discovered in humans in Belgian Congo in 1948 [6]. In the United States, it was initially identified among human isolates in the 1970s [7], and in Australia in 1978 [8]. From 1978 until 1998, there were several reports of infections that occurred outside of the United States. In Italy, this organism was recovered in 234 persons between 1980 and 1986, mostly linked to frozen eggs and egg-based products [9]. In 1996, in Australia, there was an outbreak of fifteen infections associated with peanut butter consumption [8].

Since that time, between 1998 and 2008, *S. enterica* subtype Mbandaka outbreaks had been reported only four times in the United States, most commonly linked to sprouts [10]. In 1999, there was a four-state outbreak of associated alfalfa sprouts and ungerminated seeds with 87 confirmed cases [11].

In addition, there were other clusters of infections worldwide. In Tunisia, there were 100 cases reported in 1997 and 89 cases reported in 1999, with the source of contamination never identified [12]. Between 2009 and 2010, human cases were identified in Poland, which were associated with feed and poultry [6]. New Zealand had an outbreak in 2012 associated with tahini sesame paste causing 17 illnesses associated with *S. enterica* subtypes Mbandaka and Montevideo [13]. In the United States, there was an identical outbreak in 2013 with 16 cases associated with the same product [14].

In 2013, a second outbreak of infections occurred, which was associated with contact with live baby poultry from Mt. Healthy Hatcheries in Ohio. In this outbreak, 158 persons from 30 states were infected with multiple strains of *Salmonella*, including *S. enterica* subtype Mbandaka. There were 29 persons hospitalized, and no deaths were reported [15].

Most people infected with salmonellosis develop diarrhea, fevers, and abdominal cramps 12–72 hours after being exposed to the bacteria. Symptoms typically last 4–7 days, and most people frequently recover without antibiotic treatment. In some people, *Salmonella* may spread from the intestines to the bloodstream and seed other places in the body. Immunocompromised persons are at higher risk for complications to develop with severe illness [16].

Persistent infections by nontyphoidal *Salmonella* have been described. An Israeli study demonstrated that 2.2% of infections resulted in “persistent infection”, defined as 30 days or more between cultures turning positive with the same serotype. In this study, symptomatic persistent infections were significantly associated with younger age of infection, the receipt of antibiotic treatment against *Salmonella*, hospitalization due to salmonellosis, and coinfection with other enteric pathogens. Both symptomatic and asymptomatic persistent infections were associated with anemia and receipt of probiotics. The four serovars noted to be significantly higher among persistent infections included Mbandaka, Bredeney, Infantis, and Virchow. This may suggest that serotype-specific genetic or ecological factors contribute to persistent infection in humans. In addition, about 65% of patients with persistent infections experienced a symptomatic illness with relapsing diarrhea. This may indicate that persistent symptomatic relapses with this infection for months or even years due to nontyphoidal *Salmonella* are clinically distinctive [17].

Cardiac infections develop in 1–5% of patients with salmonellosis [18]. These include endocarditis, mycotic aneurysms, device-related infections, pericarditis, mediastinitis, and infection of arteriovenous fistulas.[19]. Immunocompromised patients with alterations in local intestinal mucosal immunity may attribute to easier spread of *Salmonella* from the gut to the bloodstream [18]. *Salmonella* have a predilection to adhere to damaged endothelium of the heart and arterial walls [18–20]. One Spanish study demonstrated that the risk of endovascular infection in patients with bacteremia due to nontyphoidal salmonellosis was 23%. Most patients who develop endocarditis have an underlying heart condition [19]. In our case, the patient had a higher risk of developing bacteremia as he had GVHD involving the gastrointestinal tract. He also had underlying cardiac disease, with an atrial septal defect closure and dual chamber pacemaker implantation a few years later due to sick sinus syndrome, which placed him at an elevated risk for developing endocarditis.

There are no established treatment guidelines for *Salmonella* endocarditis. Regimens using ampicillin plus gentamicin or third-generation cephalosporins alone or in combination with gentamicin have been shown to be effective. There are also cases where fluoroquinolones have been used successfully to treat a few patients with endocarditis. In recent years, third-generation cephalsporins, because they demonstrate beta-lactamase stability, have become the treatment of choice for endocarditis [19].

Complications of cardiac infections include valve perforation, valvular ring abscess, arterioventricular wall perforation, and cusp rupture [19]. Outcomes, with decreased mortality, have improved over time. In one early case series, mortality was calculated to be 28% for patients with *S. enterica* endocarditis [19]. In a second more recent series published on all *Salmonella* species causing endocarditis, the mortality rate was 13.3% [20]. This may be due to improved diagnostic modalities, potent antibiotics, and valvular surgery [19, 20]. Among endocarditis infections involving all species of *Salmonella*, in two case series, the mortality rate of patients with endocarditis managed by medical therapy alone was higher (27.2%) compared to patients managed by both surgical and medical therapy (15%) [20]. However, to date, there are no studies that have demonstrated survival benefit with early surgery [20].

Clinically, our patient responded well with the use of prolonged intravenous antibiotics. He will continue to require close monitoring for recurrent infections.

## 4. Conclusions

We report a case of *S. enterica* Mbnadanka mitral valve endocarditis associated with the recent cereal outbreak in an immunocompromised patient. This case highlights the challenges faced in treating these infections, particularly among immunocompromised hosts.
